# Supplementary material for: Atomic Force Microscopy of Poliovirus Particles After Inactivation by Chemical Methods and Accelerated Electrons
Source: Viruses. 2025 Nov 12;17(11):1498. doi: 10.3390/v17111498 (PMC12656870; doi:10.3390/v17111498)

Supplementary Materials to the Article

Atomic force microscopy of poliovirus particles after inactivation by chemical methods and accelerated electrons

Sergey V. Kraevsky *, Sergey L. Kanashenko, Alena V. Kolesnichenko, Yury Yu. Ivin, Anastasiia N. Piniaeva, Anastasiya A. Kovpak, Aydar A. Ishmukhametov, Sergey V. Budnik, Roman S. Churyukin , Oleg A. Shilov, Dmitry D. Zhdanov

S1 Gallery of AFM images of poliovirus particles after inactivation

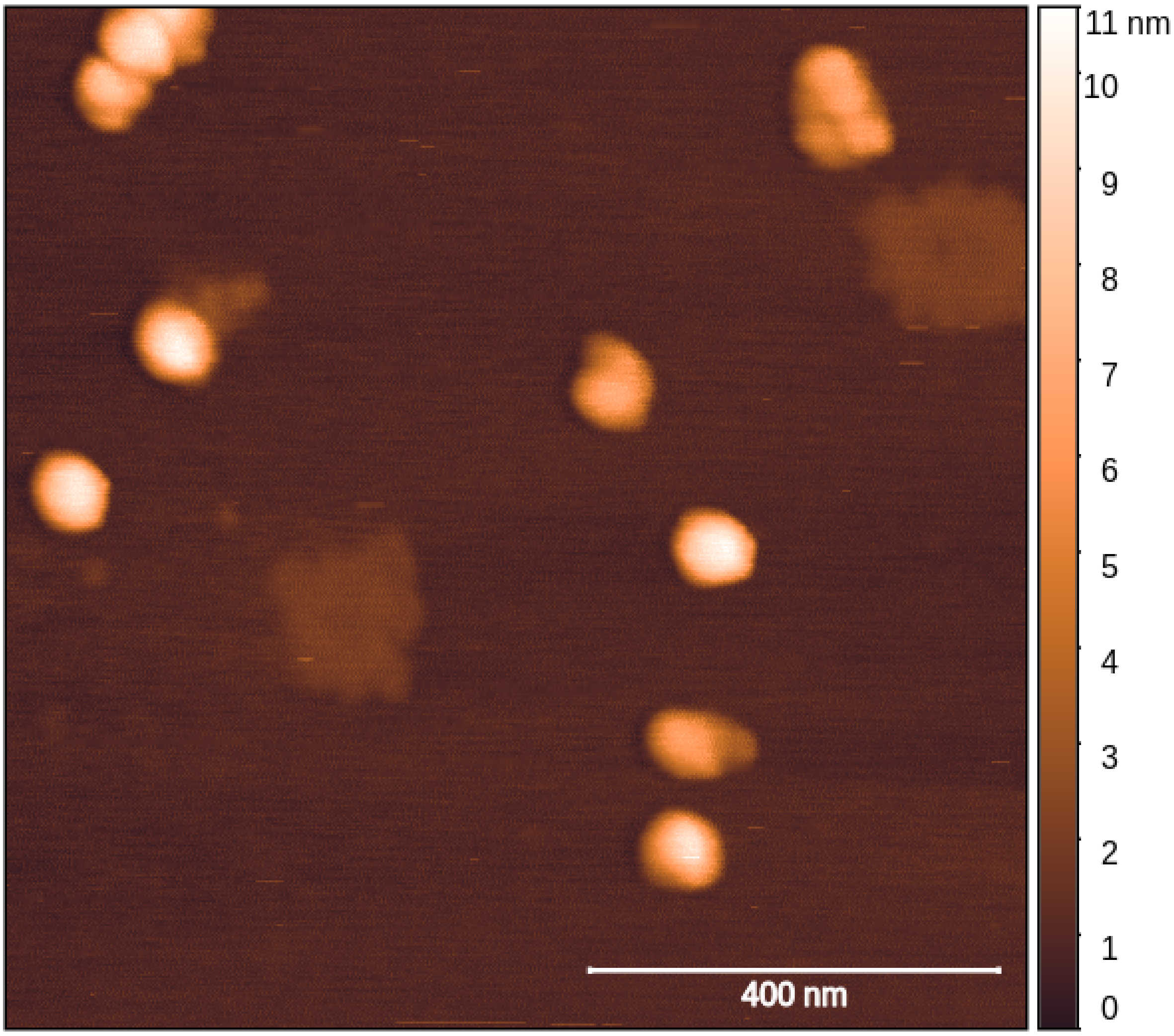

SI inactivated with β-propiolactone

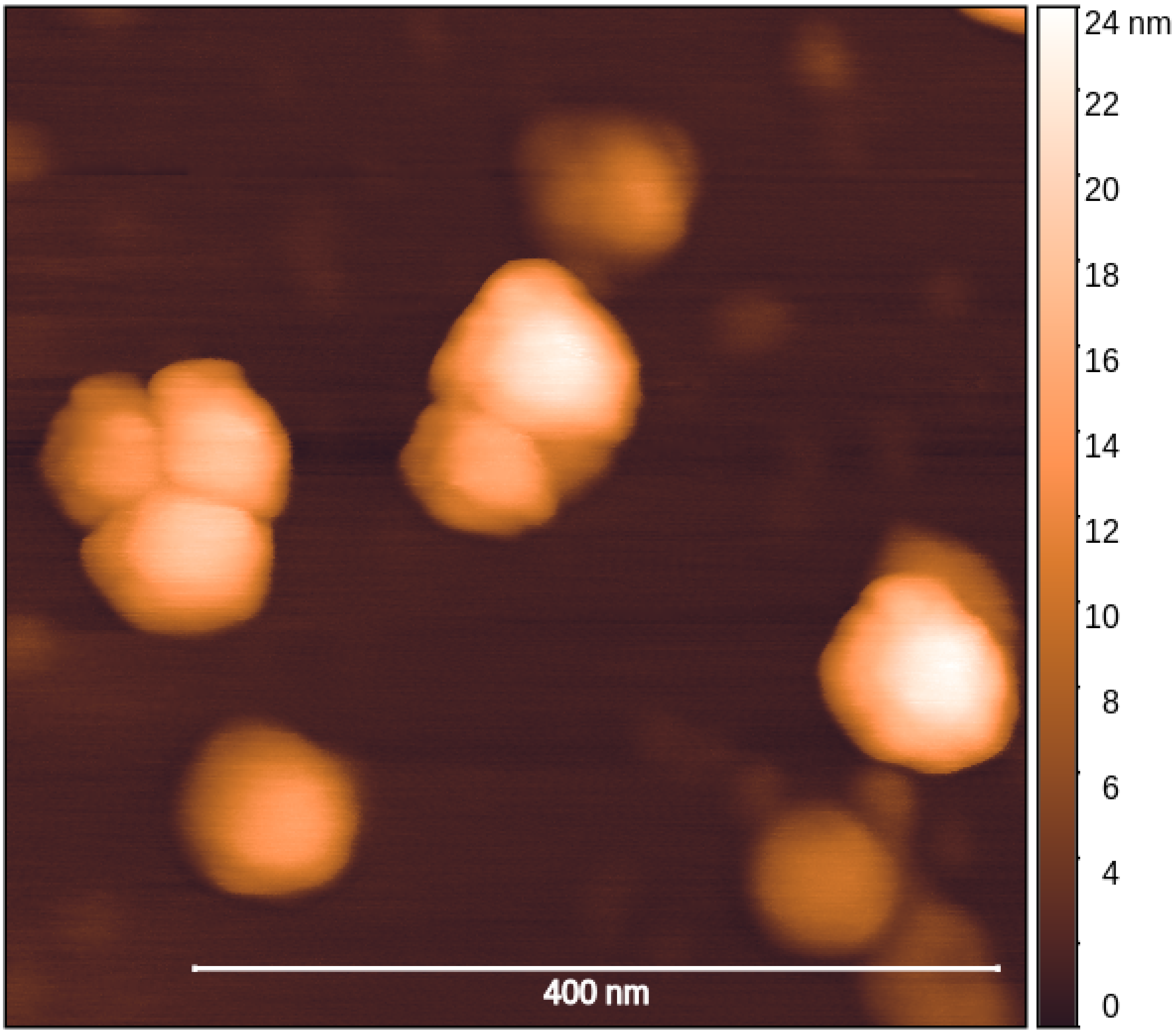

SII inactivated with β-propiolactone

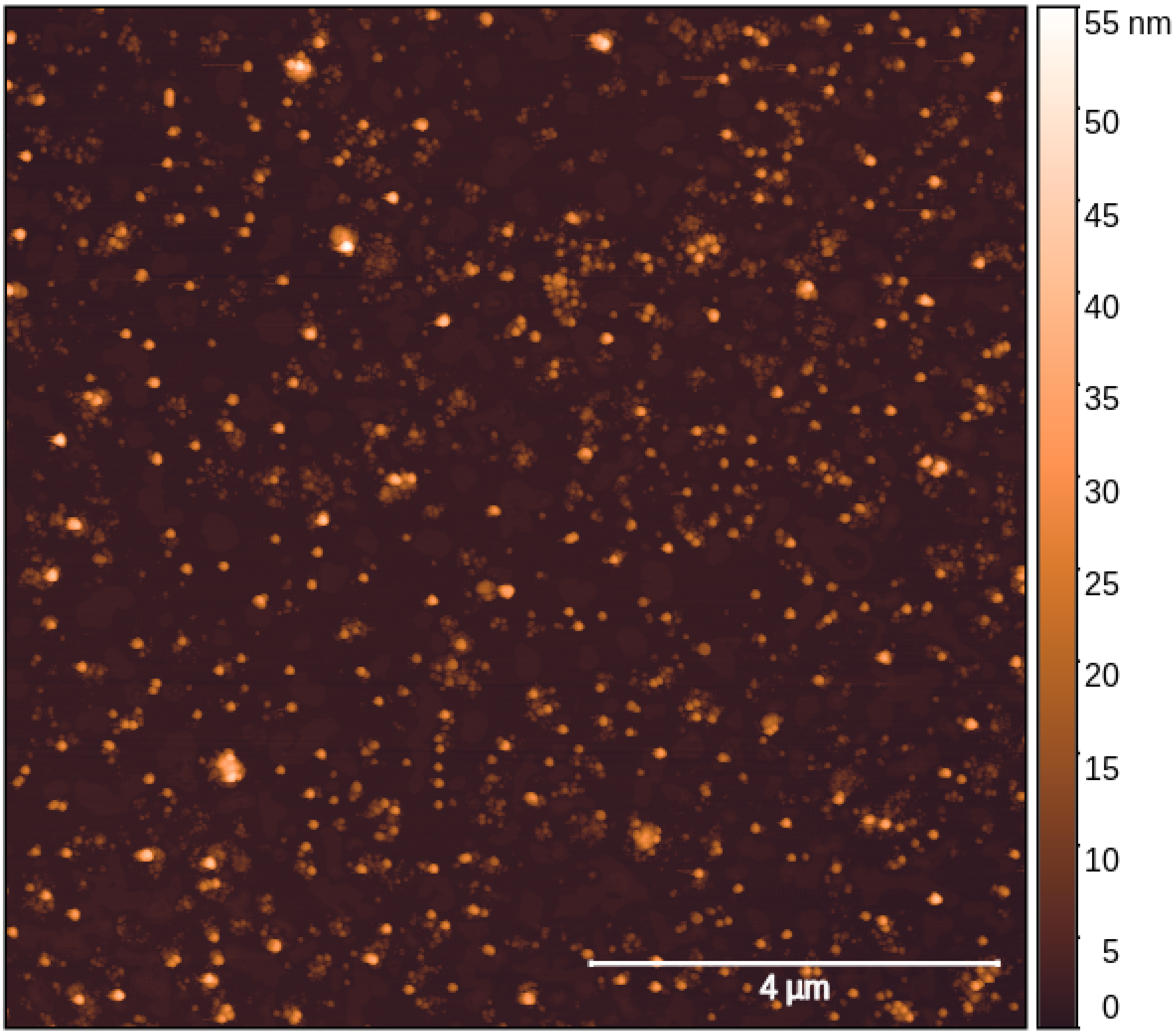

SIII nactivated with β-propiolactone

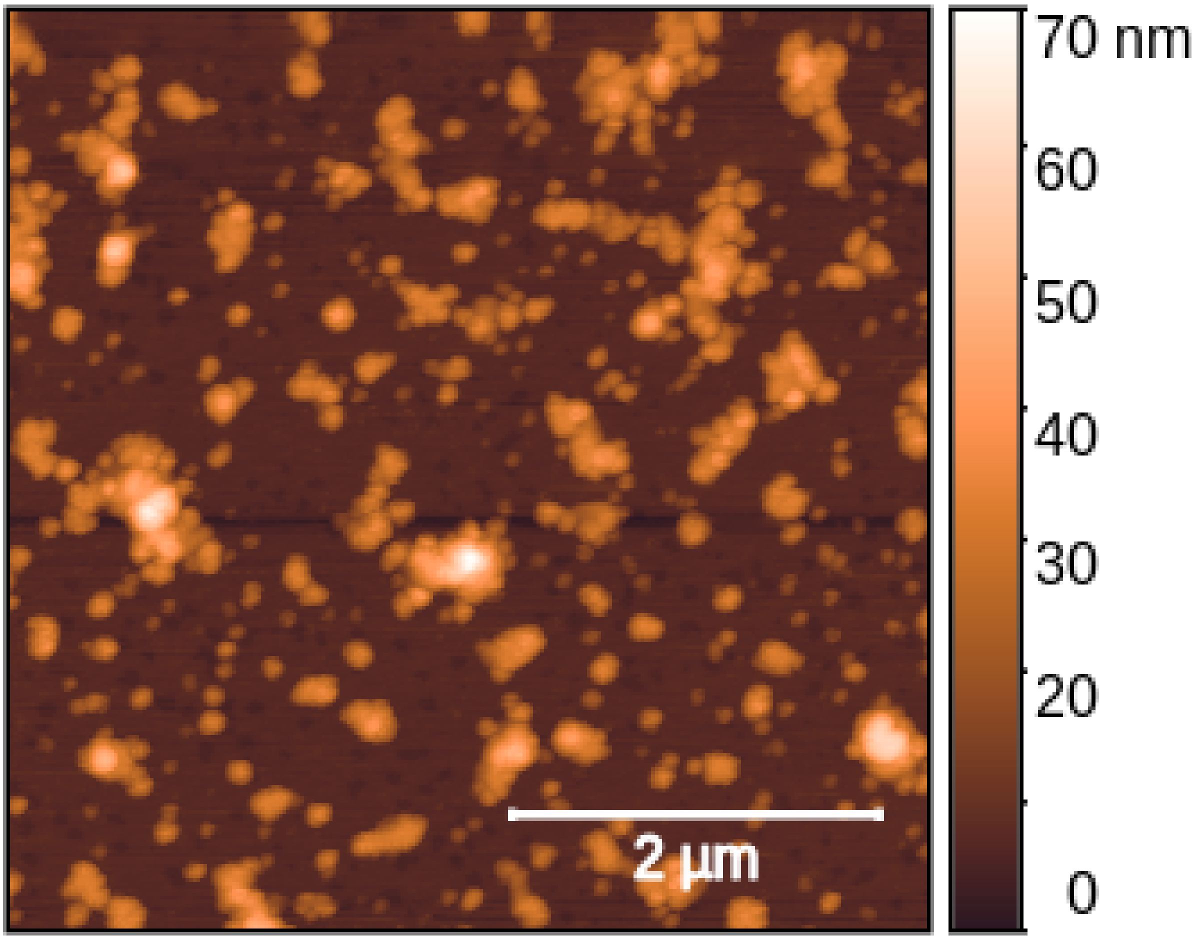

SI inactivated with formaldehyde

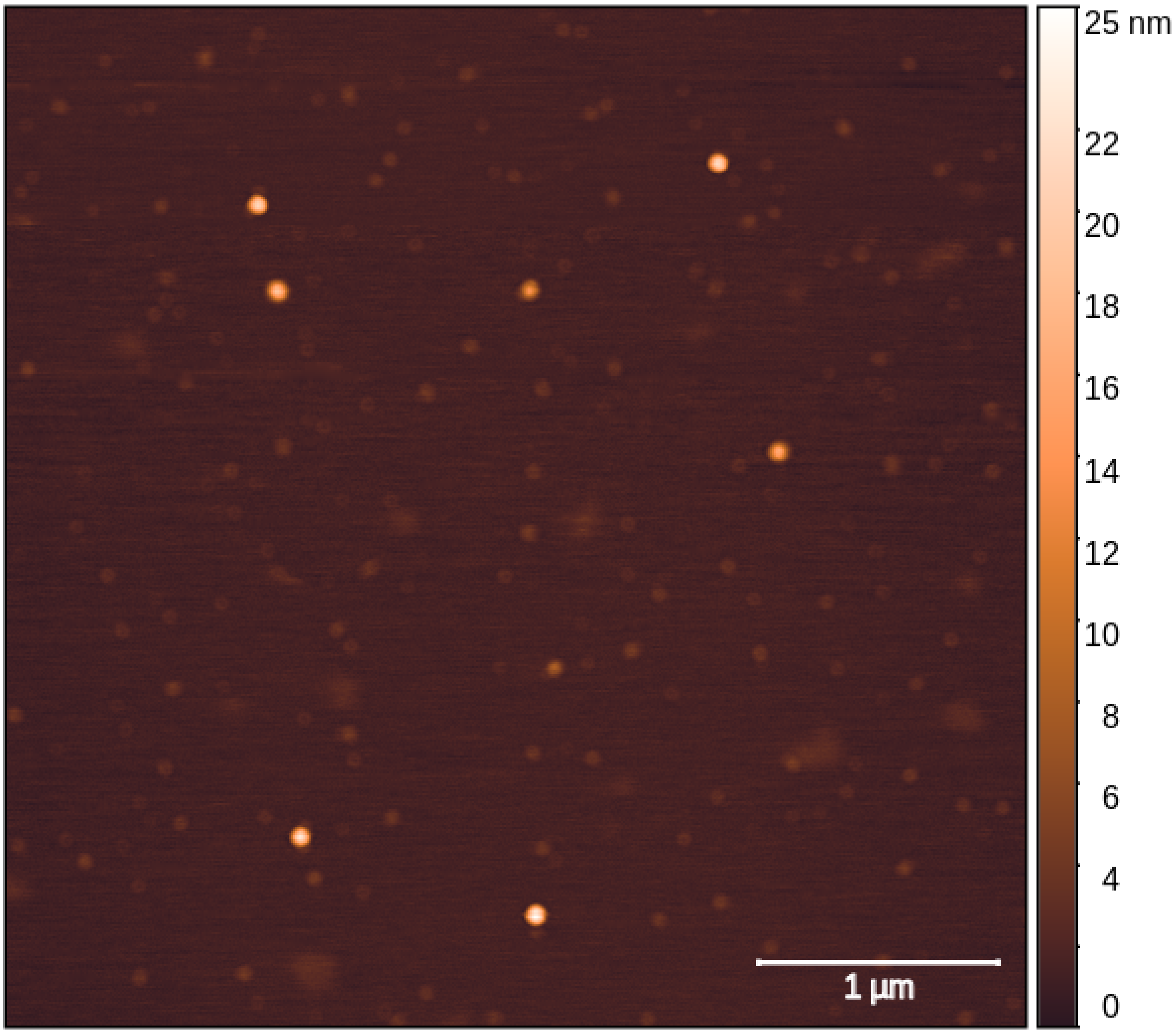

SI inactivated with formaldehyde

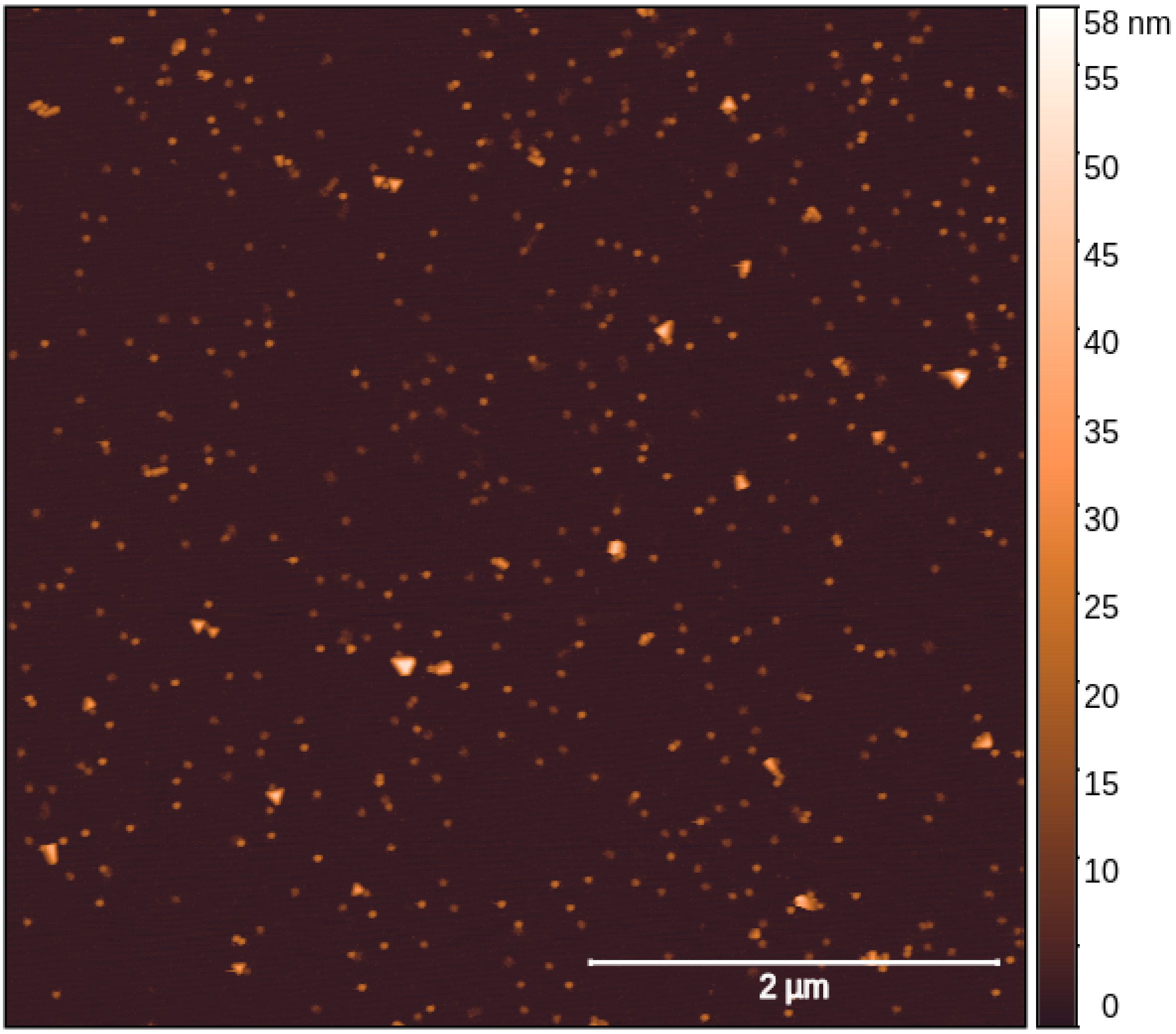

SII inactivated with formaldehyde

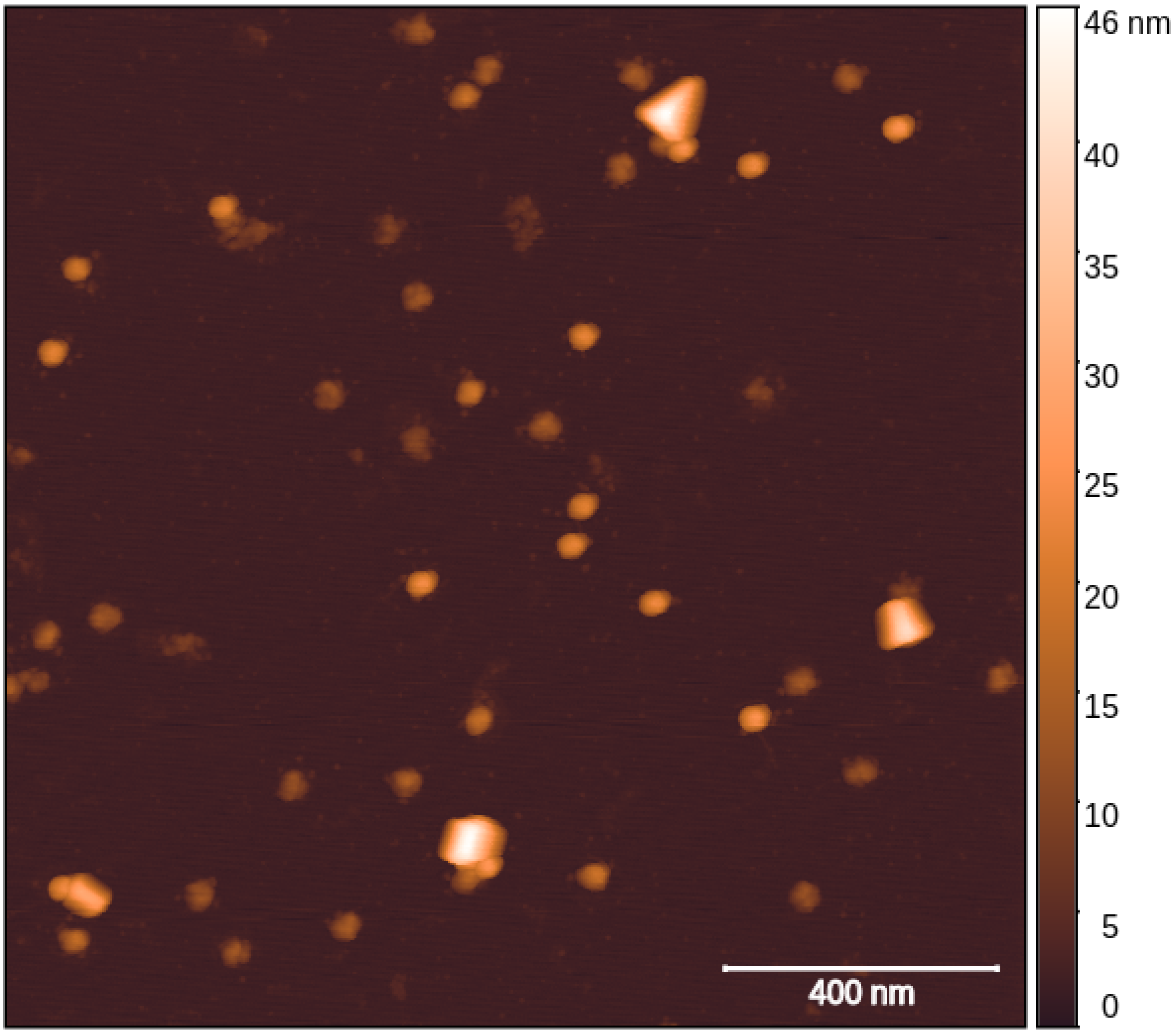

SII inactivated with formaldehyde

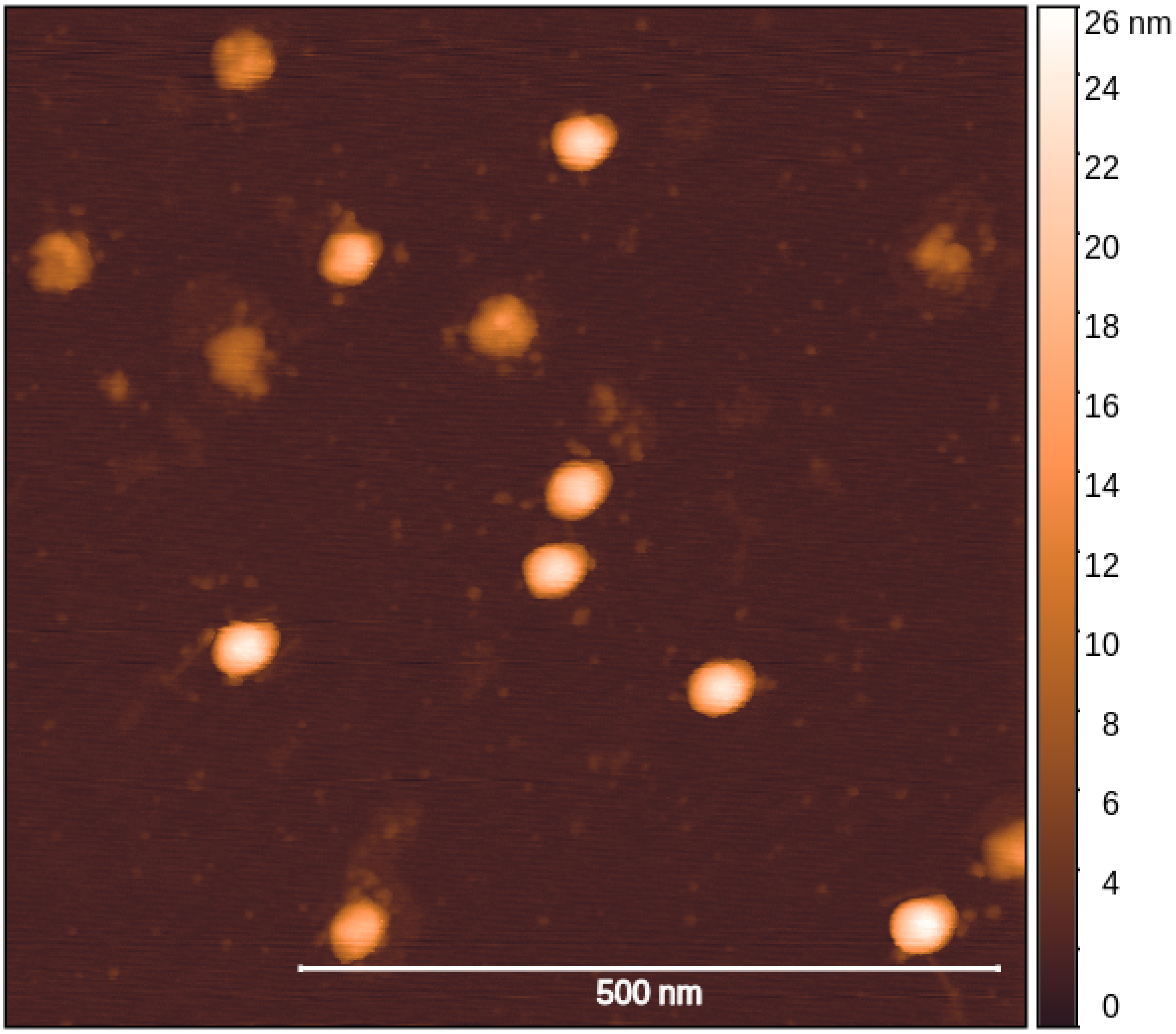

SII inactivated with formaldehyde

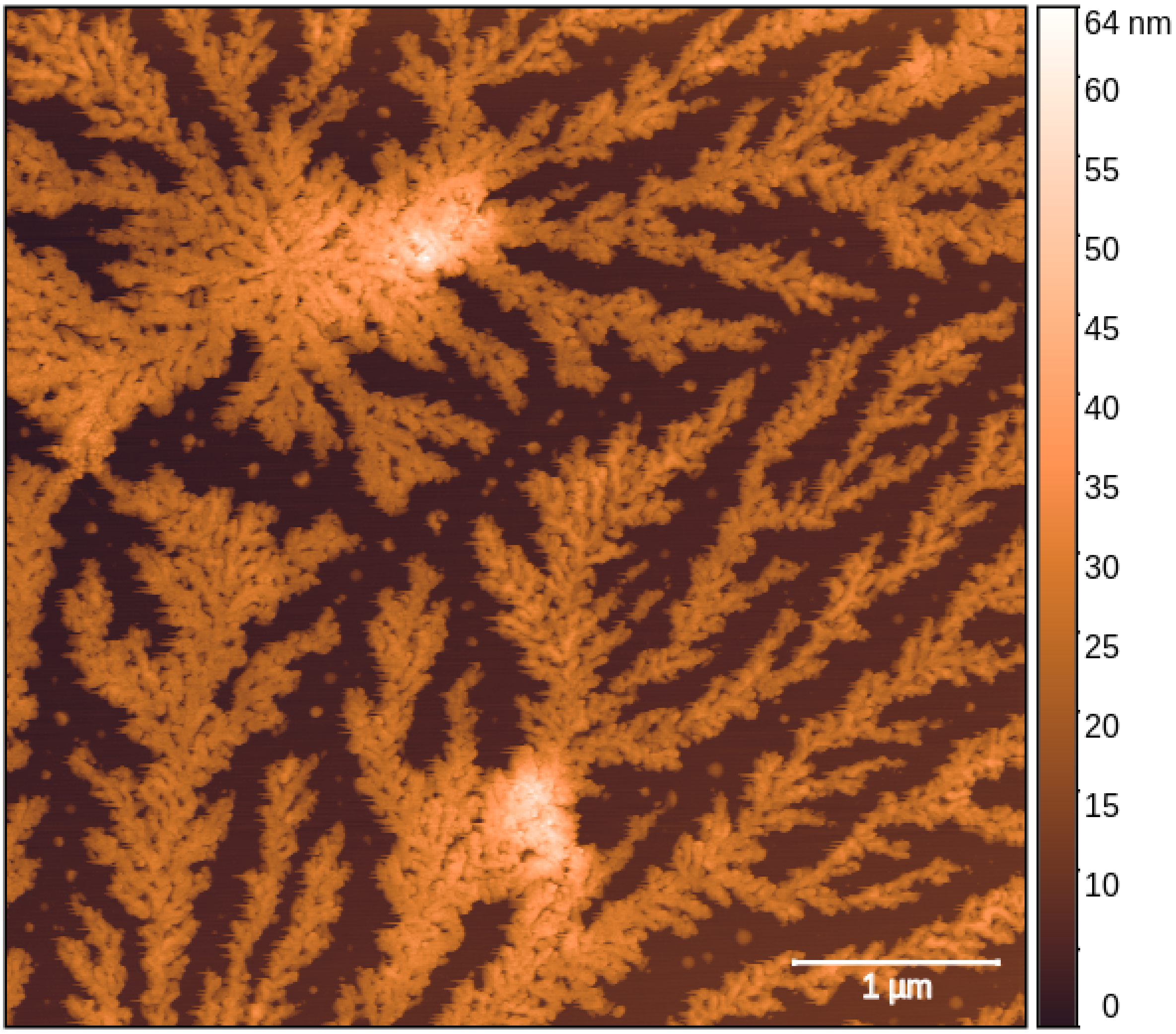

SIII inactivated with formaldehyde

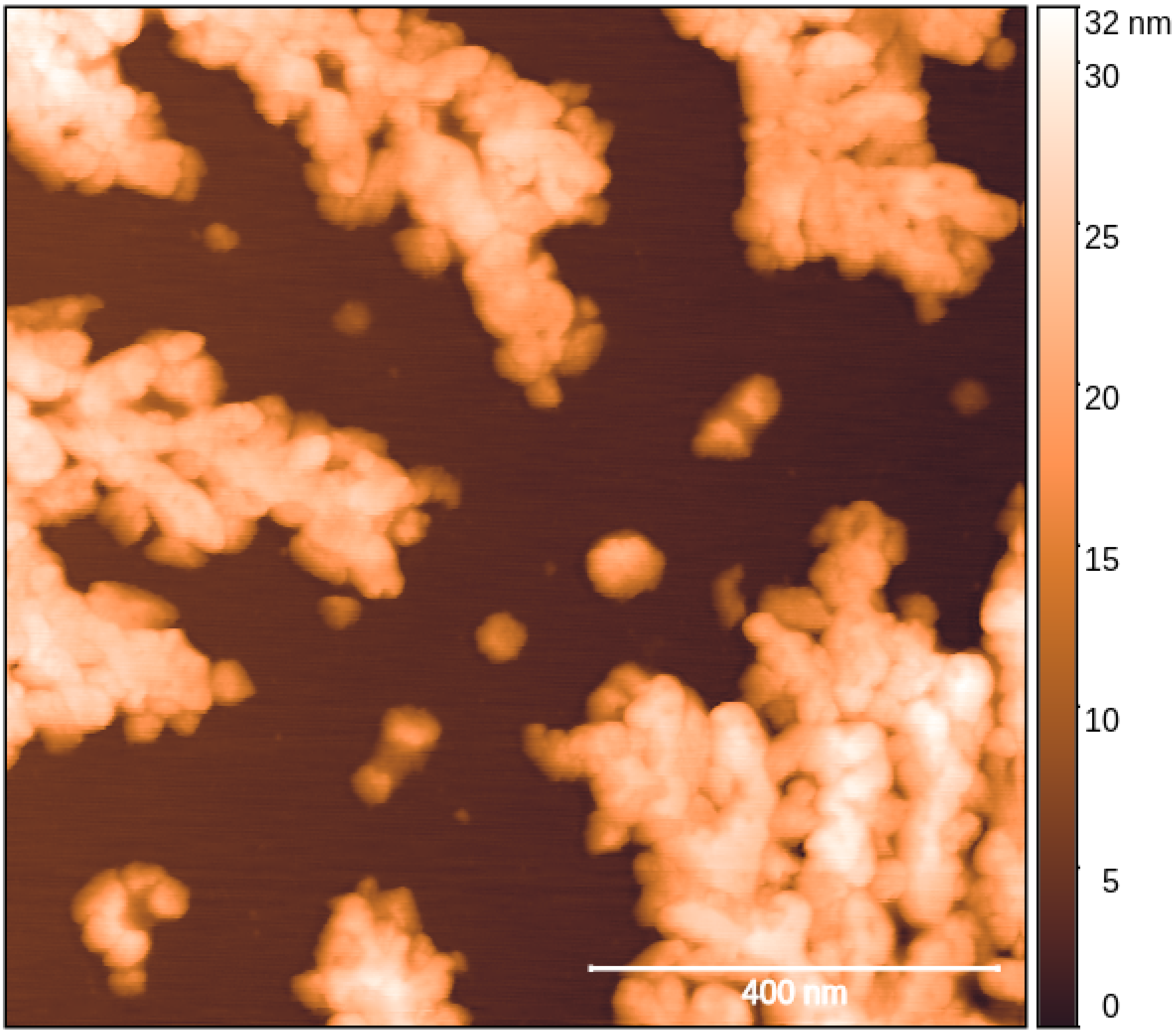

SIII inactivated with formaldehyde

S2 Gallery of AFM images of poliovirus particles SII after inactivation on Mab SII

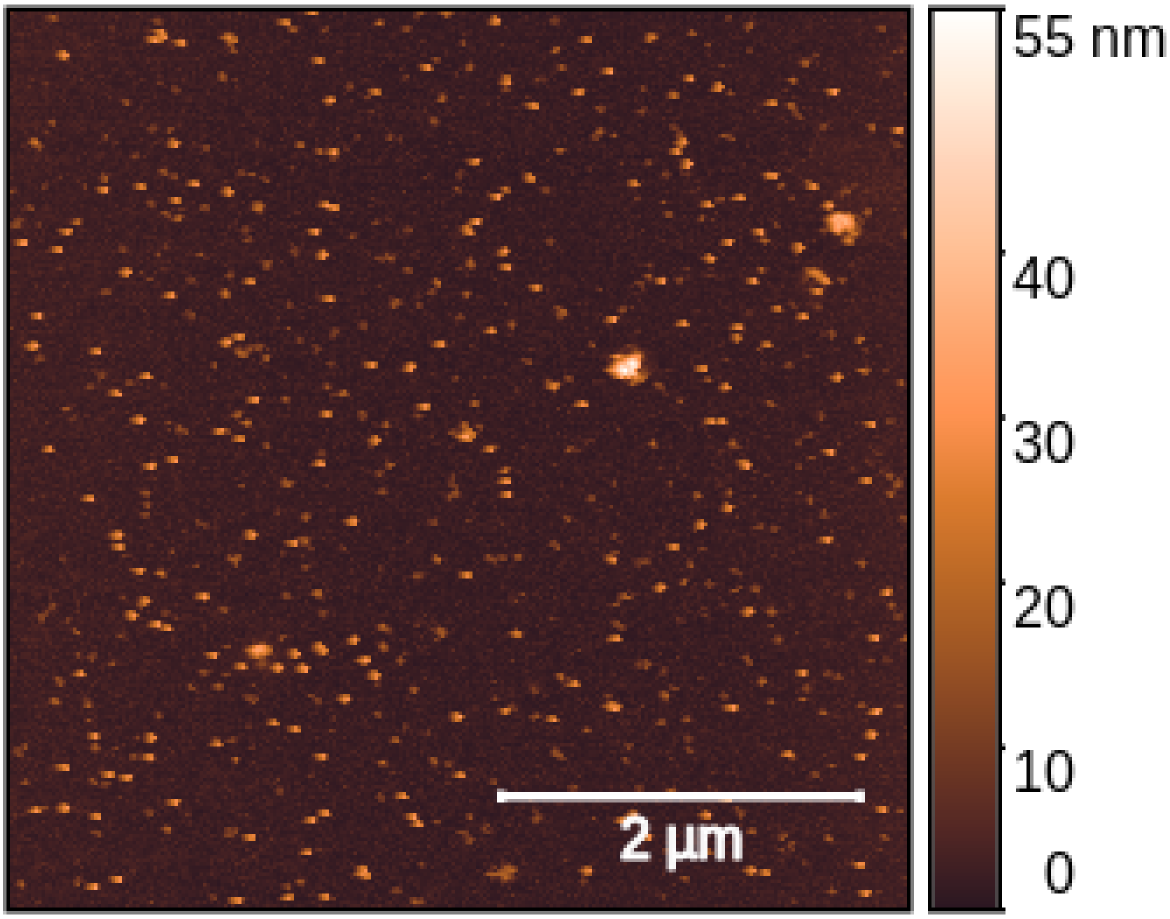

SII inactivated fast electrons

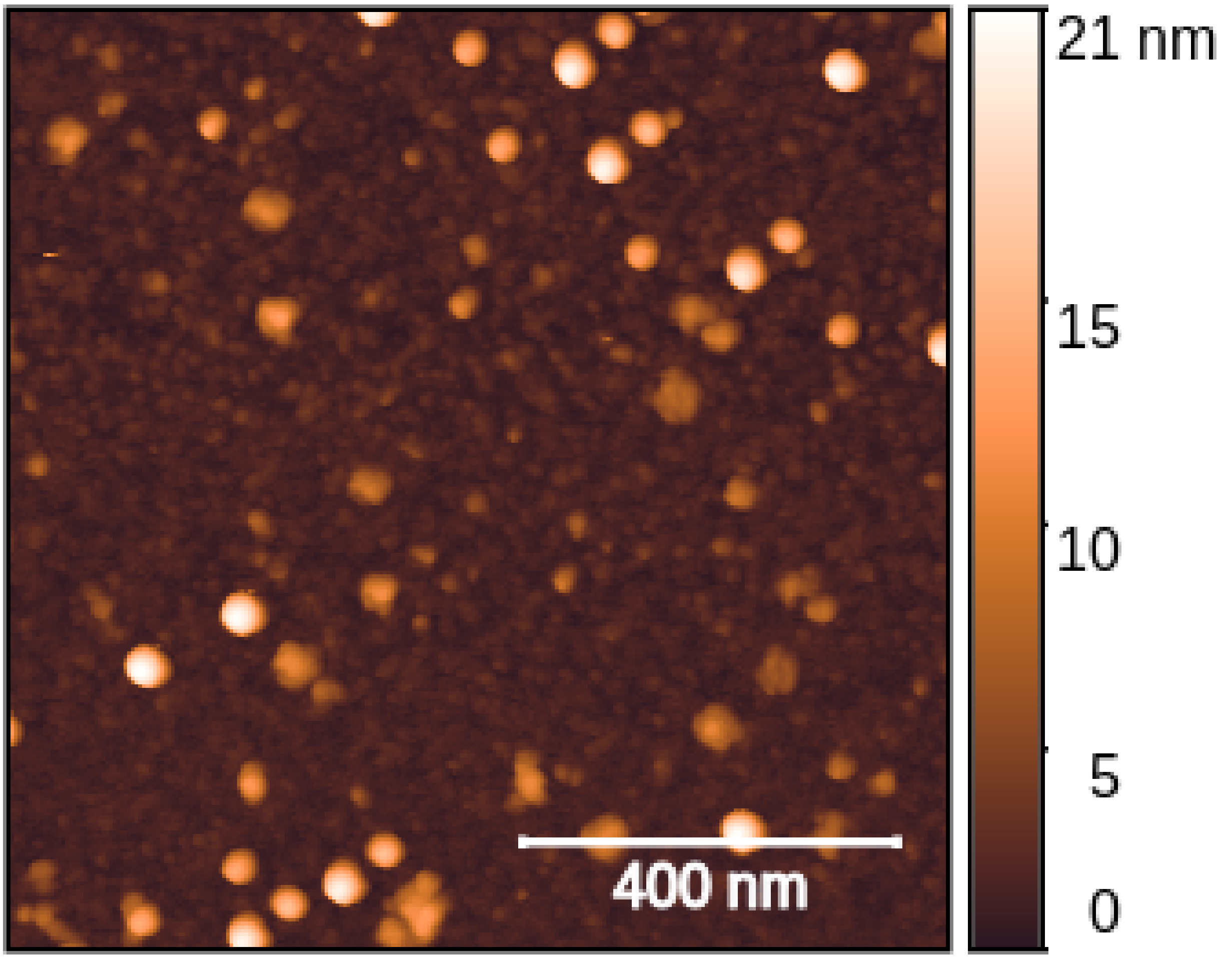

SII inactivated fast electrons

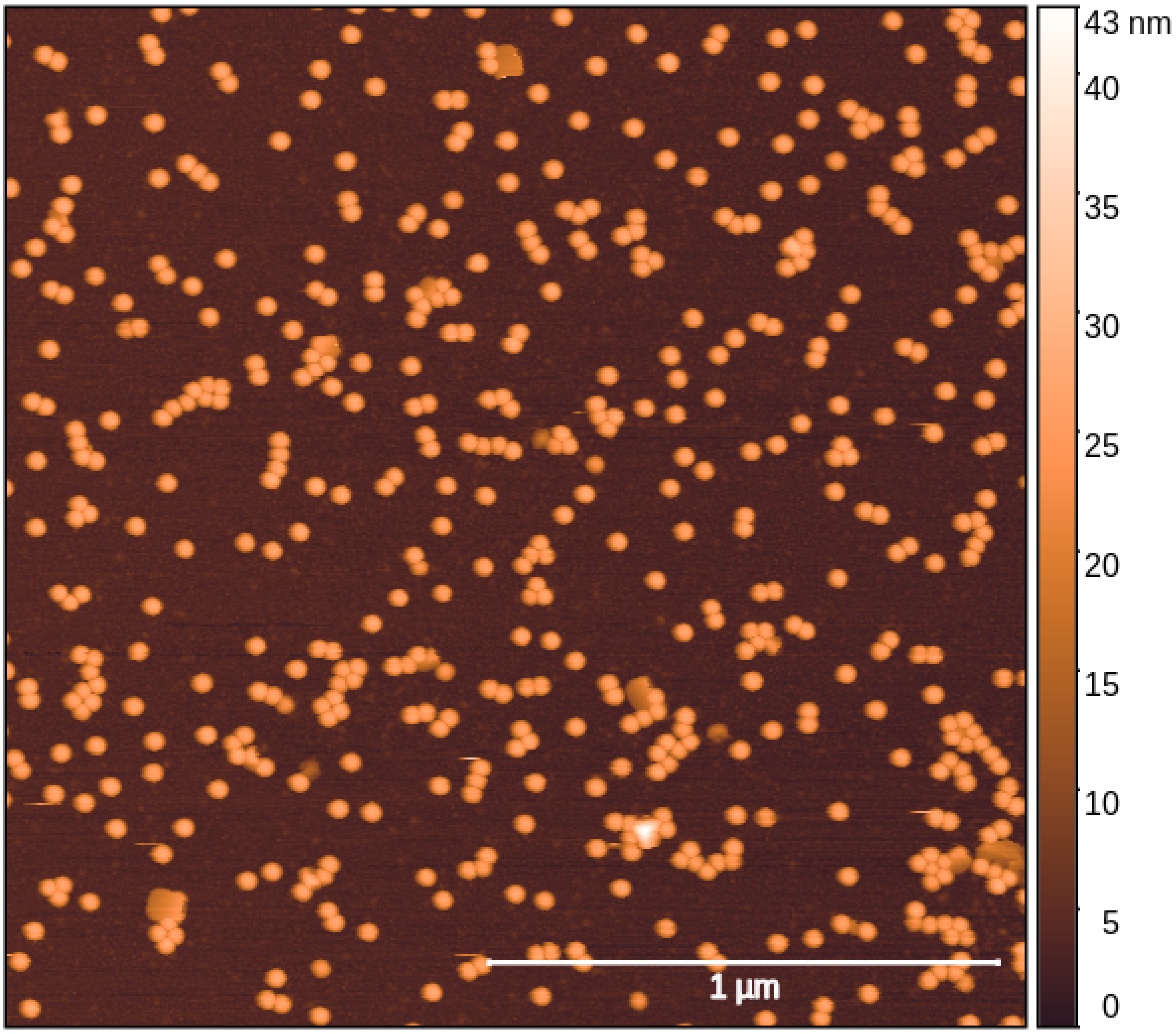

SII inactivated with β-propiolactone

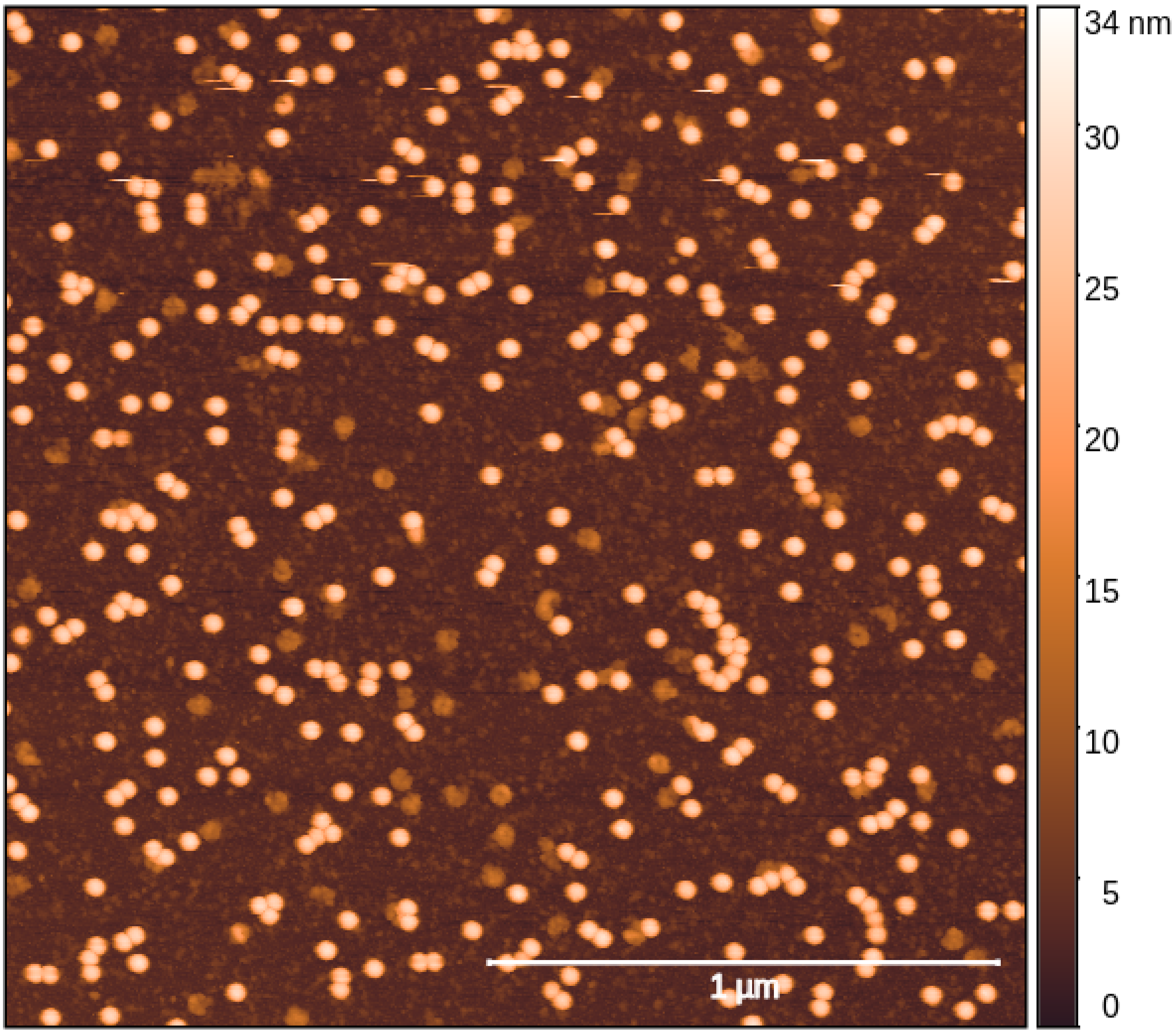

SII inactivated with formaldehyde

S3 AFM image of poliovirus particles SII aggregates inactivated with fast electrons on Mab SII after long-term storage at +4 °C

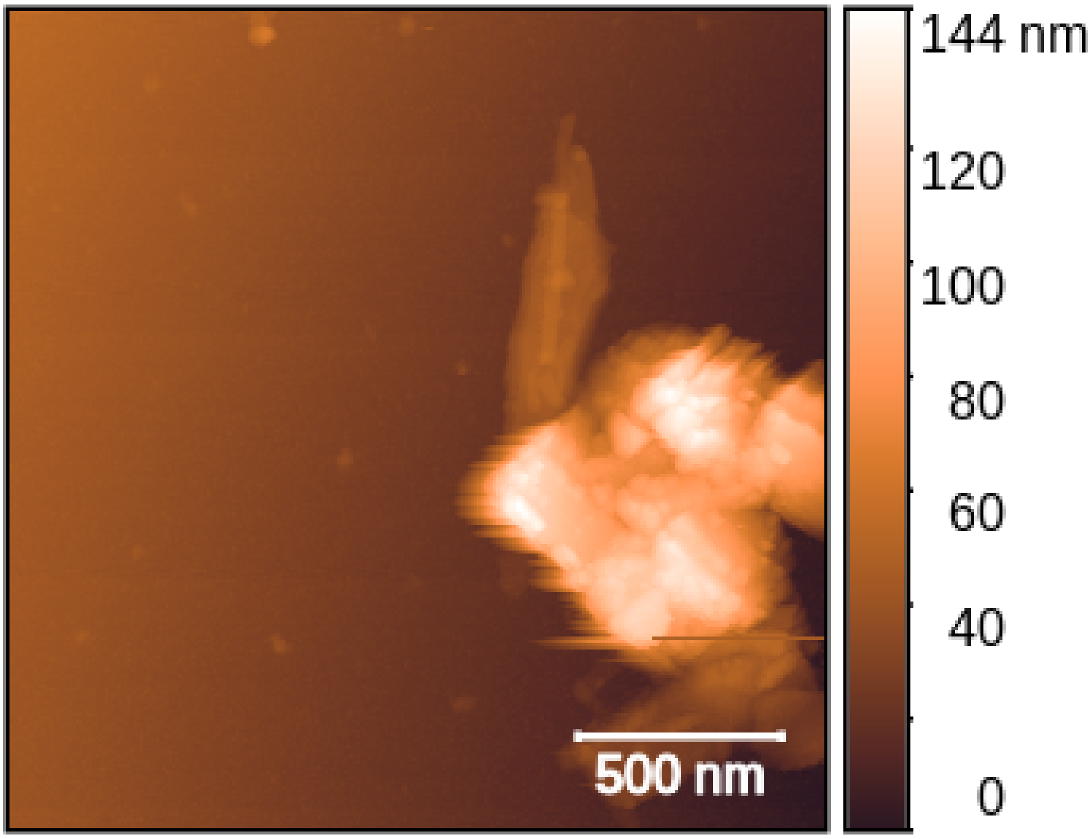

Supplement: Supplementary file 1 [file viruses-17-01498-s001.zip › viruses-3942298-supplementary.pdf]
